# Knowledge of physical activity, physical activity level and waist-to-hip ratio in adults with diabetes in a Ghanaian municipality

**DOI:** 10.4314/gmj.v57i2.5

**Published:** 2023-06

**Authors:** Adjoa N. Banson, Belinda A. Boateng, Ulric S. Abonie, Yao A. Mensah, Cosmos Yarfi, Woyram A. Kofi-Bediako, Mary W. Agoriwo, Veronica O. A. Salia

**Affiliations:** 1 Department of Physiotherapy and Rehabilitation Sciences, School of Allied Health Sciences, University of Health and Allied Sciences, Ho, Ghana; 2 Department of Physiotherapy, Tamale Teaching Hospital, Ghana; 3 Department of Nursing, School of Nursing and Midwifery, University of Health and Allied Sciences, Ho, Ghana

**Keywords:** Diabetes mellitus, exercise, obesity, knowledge, Ghana

## Abstract

**Objectives:**

To investigate the knowledge about physical activity, physical activity levels and waist-to-hip ratio among persons living with diabetes in the Ho Municipality.

**Design:**

Cross-sectional observation study.

**Setting:**

The researcher collected data from two diabetes clinics in the Ho Municipality of Ghana.

**Participants:**

Consenting persons living with diabetes who attended the diabetes clinics.

**Main outcome measures:**

Participants' waist-to-hip ratio, knowledge of the physical activity and level of activity according to the International Physical Activity Questionnaire.

**Results:**

There were 106 participants, and the modal age was 60 years or older (50.94% (n= 54)). Of the total, 62.3% (n = 66) were women, and the mean knowledge level was 12.7±1.58 (range: 0-17). Mean waist-to-hip ratio was 0.92 ± 0.10) with 25.5% (n = 27) men and 48.1% (n = 51) women recording abnormally increased waist-to-hip ratios. Additionally, 44% of participants engaged in low physical activity levels, whereas 10% participated in high levels. There were no significant associations between physical activity levels and waist-to-hip ratios (r = 0.176, p=0.071).

**Conclusion:**

Persons with diabetes in the Ho Municipality mostly engaged in low and moderate physical activity levels and had abnormally increased waist-to-hip ratios suggesting abdominal obesity. Knowledge of physical activity may be associated with physical activity performance and waist-to-hip ratio, bearing an inverse association with physical activity levels.

**Funding:**

None declared

## Introduction

Diabetes Mellitus (DM), or lifestyle diabetes, is a leading non-communicable disease globally and forms part of a disease group called metabolic syndromes.^1^,^2^ Associated morbidity and mortality figures for DM, especially over the last decade, have surged.^1^,^3^,^4^ In low and middle-income settings, DM comprises 90 to 95% of all diabetes cases.^5^ Diabetes Mellitus is characterised by hyperglycaemia and insulin resistance, resulting in poor absorption of blood glucose into body tissues (such as muscles) and depriving these tissues of the glucose needed for physical and physiologic functions.^6^,^7^ The highest risk factors for DM are lifestyle-related, namely, central obesity and low physical activity.^8^,^9^

The association between diabetes and obesity is so strong that “Diabesity” was coined in the 1970s to describe the coexistence and interrelatedness of DM and obesity. 9 Central adiposity is characteristic of abdominal obesity and is determined by a high waist-to-hip ratio (WHR). Furthermore, the World Health Organisation (WHO) has recognised that abdominal obesity is a leading risk factor for DM.^10^,^11^

Living with diabetes may be associated with potentially fatal complications such as microvascular, macrovascular, cardiovascular, and cerebrovascular diseases. Hyperglycaemia is the leading predisposing factor for diabetes comorbidities, followed by obesity and low physical activity.^6^ Additionally, time spent in sedentary behaviour is an independent risk factor for non-communicable diseases in general.^12^ It is well documented that physical activity, specifically acute bouts of exercise, reduces hyperglycaemia by absorbing blood sugar into muscles.^6^,^8^,^13^

Adults should engage in at least 30 minutes of moderate-intensity physical activity (50-70% maximum heart rate) for at least five days each week. Alternatively, 20 minutes or more of vigorous-intensity physical activity (70% or more of maximum heart rate) at least thrice weekly is recommended.^11^,^14^ Experts advise that persons with DM should engage in at least 150 minutes of moderate-intensity physical activity or 90 minutes of vigorous physical activity for a minimum of three days per week^14^ to reduce the risk of diabetes complications and improve blood sugar control.^6^,^14^

Conversely, compared with the general population, most persons with diabetes do not engage in adequate physical activity.^14^,^15^ Limited knowledge and lack of clear guidelines on the appropriate dose of exercises, 14 multiple comorbidities, little confidence in the ability to exercise, fear of hypoglycaemia 15 and lack of belief that exercise impacts the quality of life are some reported factors for low exercise patronage.^16^ Finding time to exercise and a perception that exercise is strenuous and fatiguing have also been reported, even when people with DM knew about the role of exercise in diabetes management. 13

The extent to which knowledge of physical activity relates to participation in adequate physical activity needed to reap health benefits is uncertain. Fredriksson, Alley^17^ proposed a hierarchy of ‘levels of knowledge’ regarding the benefits and risks of physical activity (or the lack thereof) for health. For example, Level 1 constitutes the knowledge that physical activity is beneficial for health and physical inactivity is harmful to health, whereas (Level 3a) indicates “knowing exactly how much physical activity is needed for health, and the probabilities of developing physical inactivity related health conditions (Level 3b)”. 17 Knowledge of physical activity is not found to predict physical activity behaviour accurately. For instance, an Australian study involving 2,535 adults found that 24% had adequate Level 3 knowledge and believed that they met national physical activity guidelines; however, their actual physical activity behaviour demonstrated otherwise. 17 Even more adults (46%) with limited physical activity participation overestimated the health benefits that the low activity level would provide them.^17^ Contrarily, a study in Canada found that more physically active people had higher knowledge of physical activity recommendations.^18^ It is important to determine how knowledge and behaviour of physical activity relate to relevant chronic conditions and diverse contexts to inform tailored paradigms and interventions.

In Ghana, increasing cases of DM have been reported;^19^,^20^, a systematic review and meta-analysis reported an overall prevalence of 6.46%. 19 However, research on knowledge of physical activity and physical activity levels among persons living with diabetes is limited. To the best of our knowledge, the research by Osei-Yeboah,^7^ is the only study in the Ghanaian setting to investigate the association between physical activity patterns and glycaemic control in persons with diabetes. The study found predominantly low physical activity participation and uncontrolled glycaemia.^7^ The current study explored the knowledge levels of persons with diabetes about the role and benefits of physical activity in DM management and how this knowledge correlates with their physical activity levels and waist-to-hip ratios. Our research findings can contribute to existing knowledge and guide treatment efforts in clinical practice to better manage diabetes and associated comorbidities through adequate performance of physical activity.

## Methods

The study was an observational cross-sectional study. All study procedures were approved by the University of Health and Allied Sciences Research and Ethics Committee (reference: UHAS-REC A.4 [306]). Permission was also sought from the participating hospitals' administrative bodies and the person in charge of the Diabetes Clinics, where persons are referred for further management and support after a medical diagnosis of diabetes. Thus, potential participants were clinic attendants for routine care during the data collection period; they were informed that the data collected would have anonymous identification and that they were free to opt-out during the study. Additionally, participants were informed that dropping out would not affect their treatment at the Diabetes Clinic.

Participants were recruited from the Ho Teaching Hospital and Ho Municipal Hospital Diabetes Clinics from January to April 2019 through public advertisements. Potential participants were provided with further details regarding the procedures and potential benefits; their concerns were addressed, and they signed an informed consent form before enrolment. The criteria for inclusion were people at least 18 years old and diagnosed with diabetes and who attended the Diabetes Clinic. Persons who had challenges that prevented them from understanding the instructions of the data collection and the questionnaire tools, such as visual, hearing disabilities or mental health illnesses, were excluded.

### Instruments for data collection

Enrolled participants were assessed through standardised and research-customised questionnaires. The assessment tool comprised four parts. Part A collected demographic information, while Part B explored their physical activity knowledge. Part B was based on recommendations from existing literature^11^,^12^,^14^,^17^ and guided by a researcher-administered tool from a similar study. 15 The questionnaire explored knowledge of physical activity guidelines and recommendations for persons with diabetes. Therefore, the study concentrated on Level 3a physical activity knowledge, which investigated participants' knowledge of the appropriate doses for physical activity performance.^17^ We chose this focus because standard knowledge of recommended physical activity doses was a more objective measure regardless of geographical or economic context. The tool assessed participants' knowledge regarding the importance of physical activity and exercise in diabetes management with a 17-item questionnaire. In Part C, participants completed the International Physical Activity Questionnaire (IPAQ), a tool that assesses their level of physical activity. Finally, participants' waist and hip circumferences were determined with a measuring tape recorded in Part D.

### Procedure for data collection

#### Knowledge of physical activity

The original tool from Hui, Hui^15^, was piloted on five persons with diabetes in the Municipality. Based on the feedback, it was modified to have a more relatable and simple language. For instance, items 12 ‘f’ and ‘g’ in the original tool were “playing a musical instrument” and “preparing meals”. Participants were required to indicate whether these were examples of physical activity; the correct answer was ‘false’ for both.^15^ These items had contextual ambiguity since some Ghanaian musical instruments and meals required intense manpower. Therefore, these items were replaced with “walking” and “moving furniture” since these were clear examples of physical activity. The researcher administered the final questionnaire, which took 30 to 45 minutes for each participant. The participants scored the questionnaire items on a scale of 1-3 (1, agree; 2, disagree; 3, I do not know). The score ranged from 0 to 17, with a higher score indicating corresponding high physical activity knowledge. Answering more than half of the questions correctly (at least 9 out of 17) was considered ‘adequate’ knowledge.

#### Physical activity level

After filling out the IPAQ Short Form, participants' responses were analysed with the IPAQ scoring and interpretation protocol guidelines. 21 The tool is internationally recognised for assessing physical activity levels. Assessment is based on activities in the previous week under four domains, based on a person's extent of walking and engagement in moderate and vigorous intensity activities. The domains include leisure time, domestic physical activity, and work-related and transport-related physical activity.

The raw IPAQ scores were converted to Metabolic Equivalent (MET) minutes per week using the MET equivalents.^21^ The MET scores were further categorised as low physical activity (< 600 MET minutes per week), moderate physical activity (between 600 and 3000 MET minutes per week) or high (vigorous) physical activity (> 3000 MET minutes).^21^

#### Waist-to-hip ratio

This was a measure of waist circumference with a tape measure at the level of the umbilicus and of the hip circumference with the measuring tape around the greater trochanter. The formula then calculated the Waist-to-Hip Ratio (WHR):

WHR= Waist Circumference/ Hip Circumference Participants were categorised as having ‘normal’ WHR (WHR < 0.85 for women; < 0.90 for men) or having ‘abdominal obesity’ when WHR was greater than 0.85 for women or 0.90 for men. 10

#### Data analyses

Descriptive statistics of mean frequencies were computed for participants' age, sex, level of education, marital status, knowledge of physical activity and physical activity level using the Statistical Package for the Social Science (IBM SPSS Statistics, version 24). Chi-square was used to compare the sex distribution across the demographic characteristics and to compare physical activity levels between demographic sub-categories. Pearson correlation analyses were performed to determine associations between knowledge of physical activity, physical activity level and WHR. The Analysis of Variance (ANOVA) test compared the WHR means within and between the demographic sub-categories. A p-value lower than 0.05 was regarded as statistically significant. The number of participants per activity level was represented in figures and percentages. The 22 (23.3%) questionnaires which had been incompletely filled were eliminated from the inferential analysis for physical activity level.

## Results

### Demographic Profile

There were 106 recruited participants (66 women, 62.3%) and 97% of whom were at least 40 years old. Most participants (50.9%) were in the 60 to 70 age bracket, and 95.3% had formal education. The details of the demographic characteristics of the participants are represented in Table 1.

**Table 1:** Sex distribution across the demographic characteristics

Demographic characteristics	Demographic sub-categories	Sex		Frequency n(%)	Chi-square	p-value
		Men	Women			
**Age**	20-39	0	3	3(2.8)	3.391	0.183
	40-59	16	33	49(46.2)		
	60-79	24	30	54(50.9)		
**Educational Status**	No School	2	3	5(4.7)	4.374	0.224
	Primary	19	42	61(57.5)		
	Secondary	10	7	17(16.0)		
	Tertiary	9	14	23(21.7)		
**Marital Status**	Single	6	26	32(30.2)	8.293	0.016
	Married	33	40	73(69.9)		
	Divorced	1	0	1(0.9)		

### Knowledge level

The results also indicated that 44.3% of participants answered that engaging in physical activities only on weekends was sufficient to achieve health benefits, whereas half (50.9%) disagreed. Also, while 58.5% agreed that a single session of 30 minutes of physical activity was equally beneficial as breaking the activities into ten-minute bouts throughout the day, 31.1% disagreed, and 10.4% did not know the answer. The mean knowledge level was 12.68 ± 1.58 out of 17. Details of participants' knowledge of physical activity are presented in Table 2.

**Table 2:** Distribution of responses to the knowledge of physical activity questionnaire

Question Number	Questionnaire Item	Correct Answers	Choices of response		
			Agree n* (%)	Disagree n (%)	Do not Known (%)
***Knowledge of fundamental concepts***					
**1**	People should get 30 minutes of moderate physical activity on most days of the week	True	103 (97.2)	3 (2.8)	-
**2**	To engage in vigorous physical activity for three hours once in week is adequate to for the best experience of health benefits	False	42 (39.6)	54 (50.9)	10 (9.4)
**3**	Performing physical activities only on weekends is enough to achieve health benefit	False	47 (44.3)	54 (50.9)	5 (4.7)
**4**	Vigorous levels of physical activity are required by the human body for health benefit	True	75 (70.8)	27 (25.5)	4 (3.8)
**5**	Ten minutes of physical activity three times a day provide same health rewards as single bout of 30 minutes exercise at similar intensity	True	62 (58.5%)	33 (31.1)	11 (10.4)
***Physical activity and diabetes***					
**6**	Being a physical active person helps to control a person's diabetes	True	97 (91.5)	3 (2.8)	6 (5.7)
**7**	Persons living with diabetes should participate in physical activity for a minimum of 3-5days a week	True	97 (91.5)	2 (1.9)	7 (6.6)
**8**	Persons with diabetes should perform strength training	True	79 (74.5)	15 (14.2)	12 (11.3)
***Knowledge on practical physical activities***					
**9**	Dancing is a physical activity that provides health benefits	True	100 (94.3)	4 (3.8)	2 (1.9)
**10**	Riding a bicycle is a physical activity that provides health benefits	True	92 (86.8)	5 (4.7)	9 (8.5)
**11**	Weightlifting is a physical activity that provides health benefits	True	99 (93.4)	5 (4.7)	2 (1.9)
**12**	Performing household chores e.g. cleaning is a physical activity that provides health benefits	True	100 (94.3)	3 (2.8)	3 (2.8)
**13**	Walking is a physical activity that provides health benefits	True	106	-	-
**14**	Jogging is a physical activity that provides health benefits	True	105 (99.1)	1 (0.9)	-
**15**	Moving furniture is a physical activity that provides health benefits	True	73 (68.9)	23 (21.7)	10 (9.4)
**16**	Engaging in Aerobic classes is a physical activity that provides health benefits	True	104 (98.1)	1 (0.9)	1 (0.9)
**17**	Swimming is a physical activity that provides health benefits	True	100 (94.3)	2 (1.9)	8 (7.5)

* n: number of participants

### Level of physical activity

**Figure 1:**
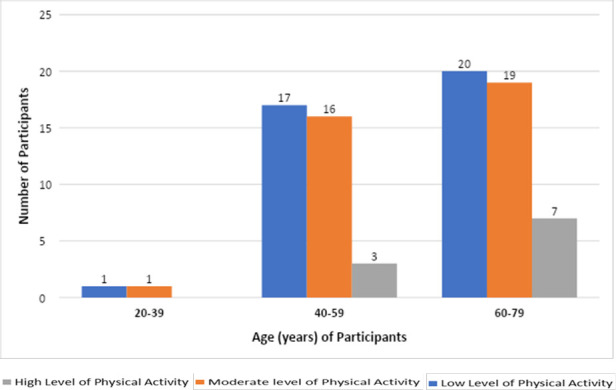
Physical activity levels of participants across age (years) categories

The study found that 44.0% (n=37), 44.0% (n=37) and 12.0% (n=10) participants engaged in low, moderate and high levels of physical activity, respectively. Persons 60-70 years of age engaged the most in moderate (23.8%) and vigorous physical activity (22.6%), whereas none of the youngest participants engaged in vigorous physical activity (See Figure 1).

Concerning sex and physical activity levels, 23.8% (n=20) more women engaged in low activity than men 20.2% (n= 17). Similarly, 29.8% (n=25) of women recorded moderate physical activity engagement than men, 14.3% (n=12). However, there were equal numbers of 6% (n=5) of men and women engaged in high levels of physical activity.

### Waist-to-Hip Ratio

The mean waist-to-hip ratio for all the participants was 0.92 ± 10. According to the ANOVA, there was no variance or significant difference in mean WHR across the demographic groups of sex, age, educational status and marital status or within each sub-category (Table 3). However, the findings indicated that 25.5% (n=27) men and 48.1% (n=51) women had waist-to-hip ratio values above 0.9 and 0.85, respectively. The WHR findings also indicated that 73.6% (n= 78) of the participants had abdominal obesity, and of the 106 participants, only 14.2% (n= 15) women and 12.3% (n= 13) men had normal body weight.

**Table 3:** Waist-to-Hip Ratio and Knowledge of Physical Activity across demographic groups

Demographic Groups	Demographic Subgroups	Mean (^†^SD)	Number (n)	p-value	
**Sex**	Men	12.8 (2.1)	40	0.682	
	Women	12.6 (1.5)	66		
**Age**	20-39	13.3 (1.5)	3	0.498	
	40-59	12.8 (1.5)	49		
	60-79	12.5 (2.0)	54		
**Educational Status**	No School	12.2 (1.5)	5	0.608	
	Primary	12.5 (1.8)	61		
	Secondary	13.1 (1.5)	17		
	Tertiary	12.7 (1.9)	23		
**Marital Status**	Single	12.6 (1.8)	32	0.155	
	Married	12.6 (1.8)	73		
		16.0 (0)	1		
***Mean Waist-To-Hip Ratios***					
**Demographic Variable**	**Description**	**Mean WHR (SD)**	**Number (n)**	**F-ratio (ANOVA)**	**p-value**
**Sex**	Men	0.92 (0.10)	40	0.066	0.797
	Women	0.91 (0.12)	66		
**Age**	20-39	0.89 (0.04)	3	0.285	0.753
					
	40-59	0.91 (0.09)	49		
	60-79	0.92 (0.12)	54		
**Educational Status**	No School	0.92 (0.16)	5	0.142	0.935
	Primary	0.92 (0.11)	61		
	Secondary	0.91 (0.09)	17		
	Tertiary	0.91 (0.10)	23		
**Marital Status**	Single	0.91 (0.12)	32	0.439	0.646
	Married	0.92 (0.10)	73		
	Divorced	0.99 (0.00)			

† SD- Standard deviation

### Associations between knowledge of physical activity, physical activity level and waist-to-hip ratio

No significant association was observed between knowledge and physical activity levels (r=0.161, p= 0.143); we found a weak positive association. Also, there was no association between knowledge level and waist-to-hip ratio (r=0.006, p= 0.949). A weak negative correlation existed between the physical activity level and Waist-to-Hip Ratio (r= -0.176, p= 0.109), but this association did not reach statistical significance.

## Discussion

This study investigated physical activity knowledge, physical activity levels, and the waist-to-hip ratio of persons living with diabetes in the Ho municipality of Ghana. We additionally investigated associations between these variables and how the physical activity levels compared with existing physical activity recommendations. Generally, participants' knowledge of physical activity was adequate. However, this knowledge did not mirror objective determinants of physical activity and obesity as measured by waist-to-hip ratio. Our findings corroborate that of Osei-Yeboah, Owiredu^7^, that low physical activity participation was common among persons living with DM in the Ho municipality of Ghana.

### Demographics

There are increasing reports of DM among people younger than 40 years old, mostly of African descent. 22 In Central Africa, compared to Caucasians, a 2-10 times increased risk of developing DM has been documented. 5 In the current study, however, most participants were aged 60 to 70 years. Diabetes in older persons attributable to impaired glucose tolerance.^23^ The current study also recorded more women cases than men, consistent with the report that DM has women preponderance across all ethnicities, attributable to a higher prevalence of overweight and obesity in women.^22^

### Demographic profile and knowledge level

The average knowledge of physical activity was 12.7 ± 1.6 out of 17, demonstrating that participants knew about the general benefits of physical activity and its importance in diabetes management. We found no significant differences in scores between the demographic sub-categories.

It cannot be established whether the education given at the Diabetes Clinics influenced participants' responses; investigating this possibility was beyond the scope of this research.

These findings suggest that specifics of Level 3a knowledge, such as physical activity guidelines and recommendations on doses,^17^ received many incorrect responses. For instance, almost half of the participants in our study responded that single bouts of exercise per week were enough for health benefits. This view is inaccurate with expert physical activity dose-response; it is recommended that persons with diabetes should not skip moderate or vigorous physical activities for more than two consecutive days per week.^14^ More importantly, occasional physical activity bouts do not negate physical inactivity's health risks.^24^ Increased time spent being inactive increases a person's risk of developing chronic diseases regardless of the level of activities performed.^25^ In the Ghanaian population, the ‘weekend’ or ‘occasional’ exercise lifestyle is rather common.^26^,^27^ Fitness programmes on holidays and organisational anniversaries in the form of indoor and outdoor games and health walks are a regular annual trend. A public figure on such an occasion mentioned that “monthly exercises would keep the people healthy to increase productivity”^26^ and the first Saturday of every month has been designated as a ‘National Keep Fit’ day.^27^ However, such public communications do not provide sufficient information, making it difficult for ordinary persons to determine the appropriate ‘regular intervals for weeks, months or years. They can translate into suboptimal physical activity performance. The weak positive association between physical activity knowledge and physical activity level may reflect this physical activity culture.

### Physical activity

Many persons with diabetes exercise less than the recommended doses.^13^,^16^ Zhao, Ford^14^ found that 31 to 37% of persons with diabetes had not exercised in the previous month prior to their study. Limited knowledge of the benefits of exercise has been suggested as a possible factor for low levels or absence of physical activity^13^,^14^ Although the knowledge of participants in the current study was relatively adequate, it did not appear to translate into the actual performance of a physical activity, as evident in the non-significant association between knowledge of the physical activity and physical activity level.

Only 12.0% engaged in high levels of physical activity, while 44.0% had low activity levels. Our research did not investigate the complete elements of the hierarchy of physical activity knowledge levels since the questionnaire mainly explored Level 3a elements; this could have contributed to the weak association between knowledge and physical activity level.

We found that 44.0% of persons engaged in low and moderate physical activity, whereas 12.0% engaged in high physical activity. An earlier study in the same Municipality also found that 30.7% of persons with DM engaged in low physical activity, whereas 48.0% and 21.3% had moderate and high physical activity levels, respectively. 7 Outside of Ghana, comparable studies by Hui, Hui^15^ revealed that 30% of persons with diabetes engaged in low physical activity. Gordon and Nelson 13 found that 38.7%, 33.5% and 26.0% of their study population engaged in low, moderate and high activity, respectively. These results consistently demonstrate the limited engagement in adequate physical activity in persons with diabetes.

Interestingly, the levels of physical activity in the current study were similar for all education sub-categories. In a previous study, 0.7 % of persons with a university education or higher participated in a high level of physical activity, whilst 34.0% of this group engaged in low activity. Additionally, compared to those with secondary school education or higher, relatively fewer persons (lower than secondary level) engaged in lower physical activity.^15^ These mixed findings suggest that factors other than knowledge may contribute to low participation in physical activity.

### Physical activity, age and sex

It is plausible that greater proportions of elderly persons and women tend to be more active than their younger and male counterparts, respectively. In the current study, the 60 to 79-years age group recorded the highest participation in moderate and vigorous levels of physical activity. Conversely, the same age group was the least physically active, with 20 out of 106 persons recording low physical activity. The finding can partly be explained by the general dominance of this 60+ age group (50.0%) in the study.

Gordon and Nelson 13 found that the mean age of persons with DM who engaged in low activity was 60.9 years, significantly older than the average age of highly active persons (53.5 years) in their study.

Conversely, Hui, Hui 15 demonstrated that older persons had significantly higher levels of activity than younger persons. Contrary to Western populations, an active older population, according to the authors, was common in China. For example, the mean age for low physical activity was 49.3 ±10.4 years, significantly younger than the mean ages for moderate (53.0 ± 10.1) and vigorous activity (51.9 ±10.8years). 15 Our study used the highest occurring age groups (modal age) rather than an overall mean age. The average age group in the current study may not be the same as the ages in the previously reported studies.^13^,^15^ We believe that since most of our participants were between 40 and 79, the mean ages reported in the previous studies fall within our participant demographic.

Reduced self-motivation, fear of falling and difficulties associated with physical disability are reported attributable factors for physical inactivity in older persons with diabetes.^28^ Moreover, older persons are likely to be retired or unemployed, thus increasing the likelihood of sedentary behaviour. However, some authors have suggested that older persons may be relatively more health conscious and, therefore, more motivated to engage in planned health promotion activities such as exercising. 15

There was no statistically significant difference in physical activity levels between men and women in the current study, although more women were found to be low and moderately active. It is also worth noting that there were 26 more women than men; it was not unusual for women to dominate all the physical activity subgroups. While men formed 20.2% of moderate and low (14.3%) physical activity levels, women dominated these groups with 29.8% and 23.8%, respectively. Previous studies^13^,^15^ also demonstrated that men with DM were more physically active than women. Others have also suggested that women engage more in traditional gender-based tasks, such as house chores and shopping, generally categorised under moderate activity. 15

### Waist-to-Hip Ratio

In the current study, the mean waist-to-hip ratios for all demographic groups were abnormally high. According to the WHO, Body Mass Index strongly predicts general obesity, whereas the waist-to-hip ratio detects abdominal obesity. 10 Abdominal obesity is found to be a superior predictor of the risk of cardiovascular disease than general obesity. In the current study, therefore, 73.6% of the participants were generally at increased risk of obesity-related death since cardiovascular disease is the primary cause of obesity-related mortality.

Abdominal obesity is also a primary risk for metabolic syndrome, characterised by impaired glucose tolerance, insulin resistance, raised plasma triglycerides and micro-albuminuria. 2 More women in our study recorded abdominal obesity, putting them at a relatively increased risk of obesity-related morbidity and mortality compared to men.

Knowledge level had a weak positive correlation with physical activity level and a weak negative correlation with WHR; the correlations were not statistically significant. For a small segment of the persons with diabetes, their reported physical activity is inversely related to their WHR. Participants with abdominal obesity may have engaged in lower levels of physical activity and the reverse for higher levels of physical activity engagement. Long-duration of moderate-intensity physical activity is documented to prevent abdominal obesity in adults.^29^ Specifically, 200 to 400 minutes of moderate-intensity exercises result in increased lipolysis in the abdominal region due to the release of catecholamines during this dose of physical activity.^29^ Thus, the accelerated use of abdominal fatty acids during physical activity to release energy may explain the inverse association between waist-to-hip- ratios and levels of physical activity. Although 44.0% of our participants recorded moderate levels of physical activity, the dose may have been inadequate to stimulate such lipolytic activity in the abdominal region. Also, other factors, including lifestyle habits, may have contributed to our findings and require further investigation.

The oldest age group in the current study was the most obese, but women also dominated this group. Authors have attributed urbanisation and westernisation of many African communities (marked by sedentary lifestyles and high-caloric diets) as the likely cause of the increasing prevalence of obesity.^5^ There has been an increased number and use of motorised transportation, especially in urban areas.^5^ Ghanaian cities, for example, have seen an influx of affordable commercial transport: motorbikes, tricycles, buses and vans. Most people use these options rather than walking even for short distances; therefore, sedentary behaviour is reinforced.

It is also possible that other factors, such as cultural practices, may influence observed obesity in elderly persons and women and warrant further investigation. For instance, in Ghanaian culture, keeping adults, especially older persons, from engaging in physically strenuous tasks is seen as honour and respect for seniors. Typically, younger women would perform house chores so their mothers and grandmothers would not have to. It is also uncommon to see women, especially elderly ones, jogging, riding a bicycle or swimming in the typical Ghanaian community. 30 Therefore, older persons may not engage in adequate physical activity despite knowing that physical activity engagement is beneficial in managing their condition.

### Limitations

The IPAQ questionnaire is subjective and depends on the ability to recall activities from the previous week. Participants may have underreported sedentary behaviour or overestimated physical activity engagements. Thus, true physical activity performance was not determinable. Also, the knowledge questionnaire may not fully reflect participants' understanding of physical activity.

### Implications for practice and recommendations for future research

It may be important to complement physical activity education with actual exercise therapy that suits the demographic diversity of persons with diabetes by including professionals such as physiotherapists in the standard management of DM. Incorporating wearable physical activity trackers may also help persons to objectively monitor their physical activity levels, thus, improving their sense of control over their health and promoting exercise adherence.

More rigorous methods incorporating physical activity tracking can investigate the different levels of knowledge of the physical activity and factors influencing participation for the DM population in Ghana.

## Conclusion

The average person living with diabetes had adequate knowledge about physical activity; however, 44% engaged in low levels of physical activity, whilst only 12.0% recorded high levels. There were also high waist-to-hip ratios indicative of abdominal obesity. The lack of significant associations to knowledge levels, physical activity level, and waist-to-hip ratios may be researched further using more rigorous methods.
